# Editorial: 73rd annual meeting of the Italian society of physiology: advancement in basic and translational physiology

**DOI:** 10.3389/fphys.2024.1547767

**Published:** 2025-01-07

**Authors:** Maurizio Cammalleri, Paola Bagnoli, Massimo Dal Monte

**Affiliations:** Department of Biology, University of Pisa, Pisa, Italy

**Keywords:** angiogenesis, endothelial cells, retinopathy of prematurity, corpus callosum, posterior parietal cortex, torpor, hypoxia-inducible factors

The 73rd Annual Meeting of the Italian Society of Physiology, held in Pisa in September 2023, brought together physiologists from Italian universities and research centers. In the 3-day Meeting, topics covering different faces of physiology have been addressed and discussed. In the present issue, some of these arguments have been outlined by original papers or reviews depicting the current state-of-the-art in specific fields of physiology with a watchful eye on basic research and its translational application.

Although well identified in both physiological (wound healing, endometrial growth, etc.) and pathological (tumorigenesis, retinopathies, inflammatory diseases, etc.) conditions, comprehensive understanding of angiogenesis mechanisms is still under exploration and efficient treatment to counteract pathological angiogenesis has yet to be found. An essential part in angiogenesis is played by endothelial cell proliferation and migration, two processes in which the role of cytosolic calcium is paramount. The generation of calcium signals in endothelial cells is triggered by the release of calcium from the endoplasmic reticulum and maintained over time by store-operated calcium entry through the plasma membrane although the calcium stored in lysosomes may also play a key role. Preclinical evidence about major mechanisms underlying the role of endothelial cells in angiogenesis has been provided by the original paper of Brunetti et al. The authors have indeed demonstrated that, in human brain microvascular endothelial cells, the Transient Receptor Potential Mucolipin one channel (TRPML1) mediates the release of calcium from lysosomes thus participating to the increase in global calcium signaling. In turn, the global calcium increase mediated by TRPML1 leads to the production of the vasorelaxant mediator nitric oxide, thus suggesting a possible involvement of this channel in the regulation of cerebral blood flow. In physiologic conditions, angiogenesis is controlled by the balanced production of pro- and anti-angiogenic factors. When the oxygen supply to tissues becomes inadequate, the consequent hypoxia triggers angiogenesis, which participates in the adaptive responses to reduced oxygen tension. In this respect, the angiogenic response to hypoxia is mainly driven by hypoxia-inducible factors (HIFs), whose role in angiogenesis is well outlined in the review of Monaci et al. That of HIFs is a family of transcription factors responsible for the transactivation of genes involved in cell survival, cell proliferation, anaerobic glycolysis, autophagy, apoptosis and angiogenesis. As a result, HIFs alter the balance between pro- and anti-angiogenic factors setting off the production of new blood vessels, thus suggesting that HIF-activated signaling pathways may be a suitable target for therapeutic strategies aimed to counteract angiogenesis-related diseases. In this respect, retinopathy of prematurity (ROP), which affects a large number of preterm newborns, is characterized by abnormal blood vessels growing into the retina possibly leading to its detachment and vision loss. In a mouse model of ROP, the role of the beta adrenergic system has been evidenced and the use of propranolol, a nonspecific beta adrenoceptor antagonist, has proven to be an excellent strategy to reduce the angiogenesis-driven retinal damage. As extensively reviewed by Cammalleri et al., results from preclinical studies laid the ground for clinical trials in which propranolol proved to be safe and effective in preventing ROP progression when administered as eye drops, suggesting the possibility that the beta adrenergic system may be a promising target to treat neovascular diseases of the retina. The additional review of Barbaresi et al. moved to higher brain functions by focusing to the intrinsic organization of the corpus callosum, a system of commissural fibers that connects the two cerebral hemispheres thus playing a key role in bilateral sensory integration and higher cognitive functions. In particular, the authors outlined three major aspects of the callosal organization including the topographical and neurochemical organization of the intracallosal fibers, the role of glia and the role of the intracallosal neurons. Then, in the context of higher brain functions, Fattori et al. reviewed the seminal work of the research group of Claudio Galletti leading to the identification of the role of the area V6A in the posterior parietal cortex, which serves to integrate sensory with motor cues in respect to voluntary actions. This review provides a comprehensive overview of studies performed in both humans and non-human primates that, starting at the end of 1980s, have changed our knowledge about the visual function. In particular, the review highlights the role of neurons belonging to the V6A area in the interactions between the subject and objects in his environment. Finally, much information derives from the original paper of Salucci et al. who tried to identify the morphological characteristics of torpor, which, in the context of energy-saving strategies, is incredibly efficient to save energy by lowering metabolic rate, heart rate and typically body temperatures. In particular, mimicking natural torpor in non-hibernating animals by inhibiting neurons in the Raphe Pallidus, a crucial thermoregulatory nucleus in the brainstem, allowed to determine the morphological correlates of organ adaptation during an induced artificial torpor state in rats. As shown by the research article of Salucci et al., among distinct organ-specific changes associated with torpor, some appeared consistent with those noted in natural torpor, while others were unique to the synthetic torpor condition. These findings suggest the possibility to exploit, in a translational perspective, system adaptations to low-energy expenditure conditions thus providing a valuable foundation for understanding organ-specific responses.

In conclusion, we are grateful to the contributors of this Research Topic and to the colleagues who revised their works thus allowing us to collect a series of papers that can be of inspiration to those scientists who are working in the field, and to those who are approaching this fascinating Research Topic. Our acknowledgements are mostly extended to the Meeting attendees. According to the English poet John Donne “*No man is an island/Entire of itself/Every man is a piece of the continent/A part of the main*” (Devotions, 1624). The sense of scientific exchange inspired the 73rd Annual Meeting of the Italian Society of Physiology thanks to the fruitful discussion among scientists with the stimulating participation of young researchers leading so far to a fundamental contribution to the development of physiology. Since the foundation of the Italian Society of Physiology in 1947, its Annual meeting represents the greatest effort in promoting physiology in all its multifaced aspects, from classical to translational physiology. Our wish is for many more successful meetings in the future!

